# Intra-rater reliability of hip abductor isometric strength testing in a standing position in older fallers and non-fallers

**DOI:** 10.1186/s11556-018-0198-6

**Published:** 2018-08-07

**Authors:** Anne-Violette Bruyneel, Simone C. Gafner, Serge Ferrari, Gabriel Gold, Dominique Monnin, Philippe Terrier, Caroline H. Bastiaenen, Lara Allet

**Affiliations:** 1 Department of Physiotherapy, School of Health Sciences, HES-SO//University of Applied Sciences and Arts Western Switzerland, Rue des Caroubiers 25, CH 1227 Carouge Geneva, Switzerland; 2 Department of Epidemiology, Research program Functioning and Rehabilitation, CAPHRI, Maastricht University, Maastricht, the Netherlands; 3 Department of Internal Medicine Specialties, University Hospitals and University of Geneva, Geneva, Switzerland; 4 Department of Internal Medicine, Rehabilitation and Geriatrics, University Hospitals and University of Geneva, Geneva, Switzerland; 5 Clinique romande de réadaptation Suva, Sion, Switzerland; 6 Institute for Research in Rehabilitation, Sion, Switzerland; 7 Department of Health, School of Health Professions, Zurich University of Applied Sciences, Winterthur, Switzerland; 8 Department of Community Medicine, University Hospitals and University of Geneva, Geneva, Switzerland

**Keywords:** Older, Falls, Muscle strength, Hip, Abductor, Standing, Reliability

## Abstract

**Background:**

Reduced hip muscle strength has been shown to be a major factor related to falls in older persons. However, comprehensive assessment of hip abduction strength in the clinical setting is challenging. The aim of this study was to investigate the feasibility and intra-rater reliability of a quick and simple hip abductor strength test in a functional standing position.

**Methods:**

Individuals over 65 years of age were recruited from the geriatric department of a university hospital and an outpatient clinic. Thirty-two older subjects, including 16 fallers (≥1 fall during the last 12 months) and 16 non-fallers were included. Maximum voluntary isometric strength (MVIS) and rate of force generation (RFG) of the hip abductors of the right leg were evaluated in a standing position using a hand-held dynamometer. Two test-sessions were carried out. All hip strength values were normalized to participants’ weight. Reliability was determined using the intra-class correlation coefficient agreement (ICC_agreement_), the standard error of measurement (SEM) and a Bland and Altman analysis (BA).

**Results:**

All participants completed the strength tests, which took a mean 2.47 ± 0.49 min (one limb). Intra-rater reliability was higher for MVIS (0.98_[0.95–0.99]_) than RFG (ICC = 0.93_[0.87–0.97]_) for the entire sample. In the non-fallers, ICC was 0.98_[0.95–1.00]_ (SEM = 0.08 N.kg^− 1^) for MVIS and 0.88_[0.75–0.96]_ for RFG (SEM = 1.34 N.kg^-1^.s^-1^). In the fallers, ICC was 0.94_[0.89–0.98]_ (SEM = 0.11 N.kg^− 1^) for MVIS and 0.93_[0.84–0.98]_ (SEM = 1.12 N.kg^− 1^.s^− 1^) for RFG. The BA plot showed that the MVIS and RFG values did not differ across test-sessions, showing that no learning effect occurred (no systematic effect). The mean differences between test-sessions were larger and the LOA smaller in the fallers than in the non-fallers.

**Conclusion:**

Assessment of hip strength in a standing position is feasible, rapid and reliable. We therefore recommend this position for clinical practice. Future studies should investigate the diagnostic value of hip abductor strength in standing to discriminate between fallers and non-fallers, and to determine if change in strength following a falls prevention program reduces the risk of falls.

## Background

According to the World Health Organization, the proportion of people above the age of 60 years is growing more rapidly than any other age group [1]. In 2025, around 1.2 billion people worldwide will be over the age of 60. Approximately 30% of people over 65 years of age fall each year. Falls lead to injuries, deconditioning, loss of independence and quality of life, and even death [2, 3]. Sixty-five percent of falls occur indoors, with 49% occurring while walking within the home [2].

The most recent Cochrane review showed that exercise programs that focus on improving balance and strength reduce the rate and risk of falls, but it is unclear which strength exercises and training modalities are the most effective [3]. Weakness of the hip muscles has been shown to be a major factor related to falls in older persons [4]. Maintaining hip muscle strength is thus important to decrease the risk of falls and associated fractures and adverse events. Comprehensive assessment of sensorimotor function is necessary to develop more effective treatments and improve understanding of the relationship between strength and falls. Hip abductor strength has been identified as a key parameter related to fall-risk [5, 6]. Arvin et al. showed that hip abductor muscles play an important role in medio-lateral balance control in older people [5]. Proprioception of hip joint may be reduced if the hip abductors are fatigued [7]. Hip muscle fatigue is associated with increased gait variability, step-to-step asymmetry in the frontal plane and slower medio-lateral trunk motion [7], all of which have been shown to be associated with an increased fall-risk [8–10]. Addisson et al. also found an association between hip abductor strength and balance strategies in older adults [11]. They showed that subjects with low hip abduction strength use a medial step strategy to recover balance from a perturbation in the frontal plane, whereas older adults with normal hip abductor strength use a cross-step strategy, which is more stable [11].

Given the impact of weak hip abductors on dynamic stability in older persons, a systematic, valid and reliable assessment of strength is necessary. However, the measurement of hip abductor muscle strength in the clinical setting is challenging and thus very few studies assessed psychometric properties of available strength tests [12–14]. A previous study in our group highlighted the feasibility and good intra-rater reliability of a hip abductor strength test in a side-lying position in older people [13]. In healthy young subjects, Widler et al. [12], showed that this position has the most valid and reliable results compared to supine and standing positions. Moreover, hip abductor strength measured in supine position had a better test-retest reliability in healthy older subjects compared to young subjects, especially when using a belt-resisted test [15]. This higher reliability in older subjects could be attributed to a higher between-subjects strength variation than in younger persons. Moreover, Kramer et al. suggested an higher risk of compensation during the test when the subject has strong abductor strength [15]. So, the older would compensate less than the young, which would be favorable to better reliability. Thus, these two sources of variation seem to make the reliability outcome for older in that specific situation more favorable than for younger people. It is therefore likely that standing can provide a reliable test of hip abduction strength for this older population. Indeed, although measurements are often carried out in side-lying or supine, these positions are not functional as falls mostly occur while walking [2]. In addition, the test procedure in side-lying is rather complicated for routine clinical use in older patients. Assessment in standing would be more functional and provide a better reflection of real life. In addition, this position is more practical in the clinical setting. Therefore, the challenge is to find a feasible and reliable hip abductor strength test in standing for older patients with balance disorders.

Most often clinical strength tests evaluate only the maximum strength value [12, 15, 16]. In standing position, muscle strength (maximum voluntary isometric strength - MVIS) is particularly important for static tasks like keeping balance on one leg, whereas the rate of force generation (RFG) is important for safe ambulation [6, 17, 18]. RFG is considered to be a parameter of the ability to rapidly generate strength and is an important component for joint stability and postural control [19]. An increase of MVIS is not necessarily associated with an increase of RFG [20], hence the importance of using both parameters.

The purpose of this study was to investigate the feasibility and intra-rater reliability of a hip abductor strength test in a standing position using a hand-held dynamometer (HHD) in older people at risk of falls. More specifically we assessed: 1) how many participants completed the hip abductor test, 2) the time needed to complete the test, 3) test-retest reliability for the total group, and for the faller and non-faller sub-groups, 4) the standard error of measurement (SEM) as well as the smallest detectable difference (SDD), and 5) bias associated with limits of agreement analysis. When the analysis is performed for the whole group, the more heterogeneous strength values may increase the reliability. By adding the same analyses for both groups (fallers and non-fallers) with probably more homogeneous strength values that potential could lead to a decrease in outcomes (ICC) compared to the whole group we are able to receive a full picture of reliability in the whole group and in relation to both subgroups.

We hypothesized that the hip abductor strength test in standing would be feasible, rapid and have good intra-rater reliability in the whole group and the subgroups fallers and non-fallers separately (ICC_agreement_ > 0.75).

## Methods

### Participants

Subjects aged over 65 years were consecutively recruited from the geriatric department of the Geneva University Hospital and an outpatient clinic in Switzerland. Thirty-two participants, including fallers and non-fallers, underwent the isometric hip abduction strength test in standing. A fall was defined as an event that results in a person coming to rest unintentionally on the ground or floor or other lower level, not caused by a major intrinsic event or overwhelming hazard [21]. A faller was defined as a person who experienced one or more falls during the last 12 months, while a non-faller was defined as a participant who had not fallen during the last 12 months [22]. The only inclusion criterion was to be aged 65 years or over. Patients were excluded if they had a major condition that impairs balance such as central nervous system dysfunction (i.e. hemiparesis, myelopathy or cerebellar ataxia), neuromuscular disorders, except distal symmetric peripheral neuropathy (i.e. no myopathy or myasthenia gravis), or vestibular dysfunction. Subjects with severe sepsis, metastatic cancer, angina, or angina-equivalent symptoms with exercise were also excluded. To limit the risks associated with the test, subjects with sores on the plantar surface of the foot, those who had undergone joint replacement within the previous year, had non-consolidated fractures, significant musculoskeletal deformities (i.e. amputation, Charcot-type changes) or lower limb or spinal osteoarthritis were excluded. In order to avoid errors due to a lack of understanding of the test instructions, subjects with moderate or severe dementia (Minimal Mental State Exam (MMSE) < 18) were excluded.

### Ethics

The study was approved by the ethical commission in Geneva (CCER - 14-235). All participants signed written informed consent after receiving information about the study and time to make an informed decision regarding participation.

### Dynamometer

A calibrated analog dynamometer (SENSIX®, Poitiers, France) that could measure forces between 0 and 667 N with a precision of 0.002 N was used to measure hip abductor strength (N). It was coupled with the DELSYS® System (Trigno sensor, DELSYS®, INC Boston; MA) that digitalized the analog output (3.3 V) with a sampling rate of 1926 Hz and a 16-bit resolution. Use of a HHD to measure lower limb strength has been validated [23, 24], and furthermore is not influenced by the experience of the physical therapist [25].

### Examiner

The same trained physiotherapist repeated the whole test procedure for all subjects (fallers and non-fallers).

### Procedure

**Fig. 1:**
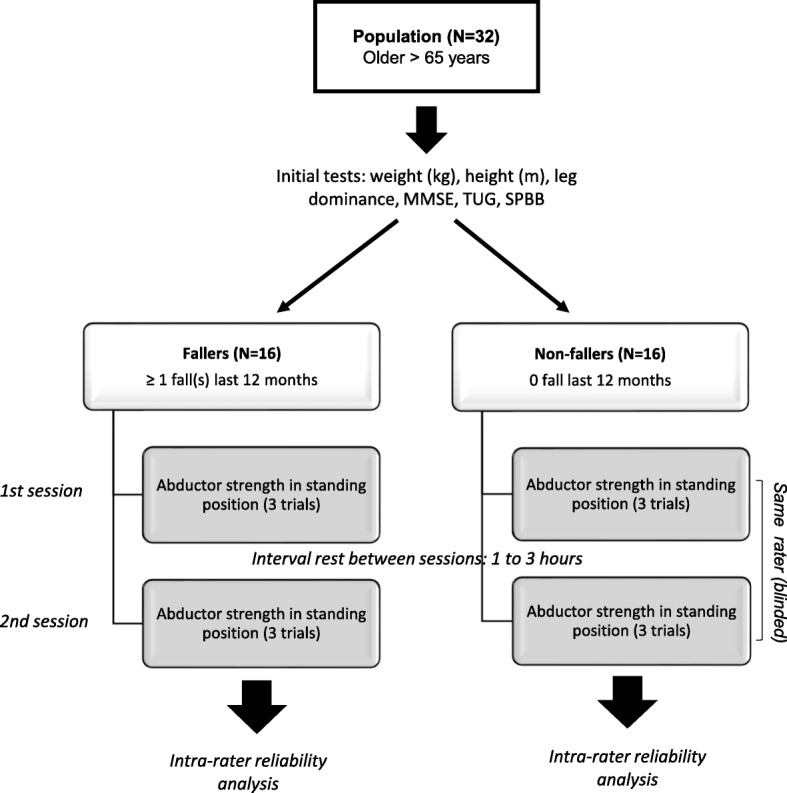
Experimental design

The procedure was carried out in 3 parts (Fig. 1): 1) clinical evaluations to characterize participants; 2) the first hip abductor strength test; and 3) the second hip abductor strength test.

The height (m) and weight (kg) of participants was measured first. Then motor function was evaluated using the Timed Up and Go Test (TUG) and the Short Physical Performance Battery (SPPB). Both tests were applied at the time of the inclusion phase, at least 1 day before the strength test to limit fatigue bias. Following this, the first strength test was carried out. In the pre-tests of this study, we compared the test repetition effect on the performance. We observed non-significant difference between tests highlighting very low learning effect. These previous results justified the absence of learning phase for these fatigable older subjects. To guarantee an identical test condition for all subjects, no pre-test participants were included in the experimental phase. So, none of the participants tried hip abductor strength testing beforehand.

**Fig. 2:**
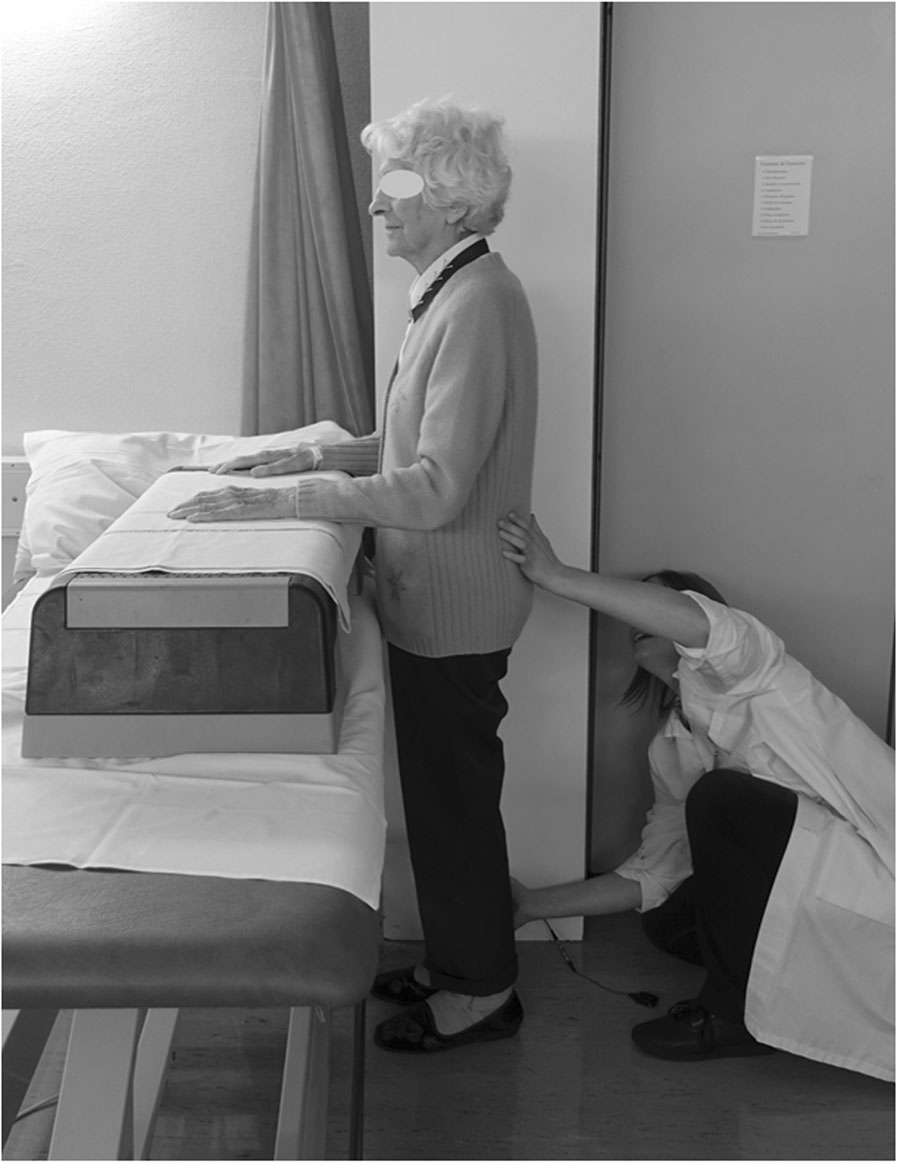
Set-up for the test

The starting position was standing side-ways-on to the wall (Fig. 2). The upper arms were by the participant’s side and the elbows were flexed to 90° with the hands resting on a treatment table adjusted to the appropriate height. Isometric hip abductor strength was measured using the HHD. To avoid biais induced by the examiner (sex and strength), the HHD was positioned against the wall, with the examiner hand. The subject’s foot didn’t touch the ground during the measurements, the knees remained in full extension throughout the test, and the tested hip was in 10° of abduction and in a neutral or slightly extended position [12, 16]. The center of the HHD was positioned on the lateral malleolus of the tested leg. The neutral hip position and the pressure on lateral malleolus induced a standardized foot position without hip compensation. The subject was instructed to push his/her leg as quickly and as hard as possible towards the HDD during 5 s. The tester’s job was to hold the dynamometer on the wall, secure the patient, give instructions and give verbal encouragement. Three tests were performed with the right leg, with a minimum 20 s of rest between tests with the foot on the ground. The test was repeated on the same day with the same examiner after a break of one to three hours. This time was estimated as being sufficient for a full recovery, but not too long for a change in performance to occur. The examiner was blind for all measurements because it was the Delsys® system that recorded measurements without the need to read the value on the dynamometer screen.

### Data processing

The raw force signals were exported to Matlab® (Mathworks®, Natick, MA, V.8.3.0.532, 2014), which was used for data processing. The signal was low-pass filtered (75 ms moving average) to attenuate high-frequency noise. Three dependent variables were extracted: test duration (s), MVIS (N) and RFG (N/s) [13, 17]. MVIS was defined as the peak value reached within four seconds, and RFG was evaluated during the 50 ms after 10% of MVIS was reached [13]. Both parameters were normalized to body mass [26]. The mean of the three trials for each test-session was calculated for the MVIS and RFG for each participant.

### Statistics

Several analyses were carried out: the number of participants that successfully completed the two test-sessions, the time needed to perform the test and, intra-rater reliability for the whole sample and for each group separately. The statistical analysis included descriptive statistics. Means and standard deviations (SD) are reported for continuous variables. A Mann Whitney U test was applied to compare test duration between-group and a Wilcoxon test was applied to compare session 1 and 2. A threshold value of *p* < 0.05 was adopted to rule out the non-significant difference. Intra-rater reliability for hip abductor strength values was determined using the ICC_agreement_ (A,1 type) model as the relative reliability index [27]. Interpretation of the ICC_agreement_ was as follows: values > 0.75 were considered as “good reliability”, between 0.5 and 0.75 as “moderate reliability” and < 0.5 as “poor reliability” [28]. In addition, we calculated confidence intervals (CI 95%) through bootstrapping (5000 resamples, bias corrected and accelerated percentile method) [13]. If the ICC_agreement_ value was above 0.50, the measurement error value using the standard error of measurement was computed: $\left(\mathrm{SEM}=\mathrm{ST}\sqrt{1-\mathrm{ICC}}\right)$ with ST being the standard deviation of all the trials of both sessions. The smallest detectable difference (SDD) was also calculated: (SDD =1.96 ∗ SEM ∗  √ 2). The SDD was normalized by the mean and expressed as percentage. Absolute reliability was investigated using Bland and Altman analysis to determine between-session agreement of the strength measurements [16, 27]. The 95% limits of agreement (LOA 95%) represent 2 standard deviations (SD) above and below the mean difference (bias) between sessions.

## Results

### Clinical characteristics

The number of potentially eligible subjects was 48. Seven subjects refused to participate (without explanation: 2, fatigue: 1, too many other exams at hospital: 2 and 2 subjects refused to sign informed consent), and 9 subjects were excluded by the investigator (too early discharge: 2, MMSE too low: 4, diagnosis: 2, poor language comprehension: 1). Finally, thirty-two older persons were included (16 fallers and 16 non-fallers). The characteristics of the whole study population as well as the characteristics per sub-group are presented in Table 1.

**Table 1:** Description in mean (±SD) of all patients and the 2 subgroups (fallers and non-fallers) recruited for the hip abduction test in standing position

Variables	All (*N* = 32)	Fallers (*N* = 16)	Non-fallers (*N* = 16)
Age (years)	83.03 ± 7.78	86.94 ± 6.23	79.12 ± 7.33
Sex (%)	Men: 34% / Women: 66%	Men: 44% / Women: 56%	Men: 25% / women: 75%
Weight (kg)	63.88 ± 12.47	59.93 ± 11.87	67.83 ± 12.13
Height (m)	1.64 ± 0.08	1.62 ± 0.08	1.66 ± 0.07
BMI (kg/m^2^)	23.62 ± 3.78	22.61 ± 3.65	24.62 ± 3.74
Leg dominance (%)	Left: 6% / Right: 94%	Left: 6% / Right: 94%	Left: 6% / Right: 94%
Number of falls (last 12 months)	N/A	3.25 ± 1.61	N/A
Walking aid (%)	Yes: 31% / No:69%	Yes: 56% / No: 44%	Yes: 6% / No: 94%
TUG (s)	15.90 ± 9.99	22.46 ± 10.33	9.34 ± 2.70
SPPB (score /12)	8.59 ± 3.50	5.88 ± 2.68	11.31 ± 1.54
MMSE (score /30)	27.06 ± 3.17	25.31 ± 3.40	28.81 ± 1.64

### Feasibility of the tests

All 32 participants successfully completed both hip abductor strength-tests. Assessment-time was less than four minutes (mean 2.47 ± 0.49 min per leg), not-including set-up time (< 1 min). For whole group, we observed non-significant (NS) difference between sessions (session 1: 166.87 ± 43.06 s vs. session 2: 167.81 ± 55.40 s, NS). The comparison of duration was NS between sub-groups for either session (session 1: non-fallers: 165.00 ± 45.17 s vs. fallers: 168.75 ± 42.25 s; session 2: non-fallers: 151.88 ± 31.88 s vs. fallers: 183.75 ± 42.25 s, NS). For each subgroup, the comparison between sessions was NS.

### Intra-rater reliability analysis

Table 2 shows the intra-rater reliability of the test using the ICC_agreement_, SEM and SDD. The total sample MVIS and RFG measures were reliable, with ICC_agreement_ above 0.75 although the ICC_a__greement_ = 0.93 _[0.87–0.97]_ for the RFG (fallers: 0.93 _[0.84–0.98]_ and non-fallers: 0.88 _[0.75–0.96]_) was lower than that of the MVIS with ICC_agreement_ = 0.98 _[0.95–0.99]_ (fallers: 0.94 _[0.89–0.98]_ and non-fallers: 0.98 _[0.95–1.00_). This was also found for the sub-groups. The SEM values for MVIS ranged from 0.11 N.kg^− 1^ (fallers) to 0.08 N.kg^− 1^ (non-fallers). For RFG, SEM values ranged from 1.12 N.kg^− 1^.s^− 1^ (fallers) to 1.34 N.kg^− 1^.s^− 1^ (non-fallers). The SDD was 32.7% for MVIS and 48.8% for RFG in fallers. SDD values were lower in non-fallers (MVIS: 20.3%, RFG: 41.2%).

**Table 2:** ICC_agreement_ (A-1), SEM and SDD (%) values for MVIS and RFG parameters of all patients and the 2 subgroups (fallers and non-fallers) recruited for the hip abduction test in standing position

	Variables	ICC_agreement_ [95%CI]	ICC_agreement_ interpretation	SEM (unit of measure)	SDD (%)
All (*N* = 32)	*MVIS*	0.98 [0.95–0.99]	Good	0.09	25.2%
	*RFG*	0.93 [0.87–0.97]	Good	1.24	44.3%
Fallers (*N* = 16)	*MVIS*	0.94 [0.89–0.98]	Good	0.11	32.7%
	*RFG*	0.93 [0.84–0.98]	Good	1.12	48.8%
Non-fallers (*N* = 16)	*MVIS*	0.98 [0.95–1.00]	Good	0.08	20.3%
	*RFG*	0.88 [0.75–0.96]	Good	1.34	41.2%

*ICC*_agreement_ intra-class correlation coefficient, *SEM* standard of error measurement, *SDD* smallest detectable difference, *MVIS* maximal voluntary isometric strength, *RFG* rate of force generation

**Fig. 3:**
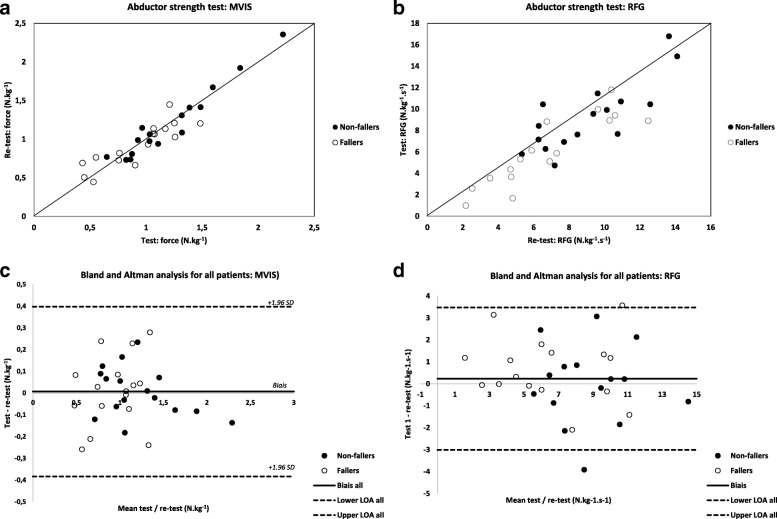
Correlations between sessions 1 and 2 for all participants and both parameters (MVIS and RFG) are represented in plots **a** and **b** respectively. The Bland and Altman plot analysis for MVIS is represented in plot **c** and the RFG in plot **d**

The MVIS and RFG values did not differ across test-sessions, showing that no learning effect occurred (no systematic effect) (Fig. 3). The mean differences were larger and the LOA smaller in the fallers than in the non-fallers, (Table 3).

**Table 3:** Bland and Altman limits of agreement between sessions for MVIS and RFG parameters of all patients and the 2 subgroups (fallers and non-fallers) recruited for the hip abduction test in standing position

	Variables	Mean (±SD) difference between sessions (biais)	Lower LOA to Upper LOA
All (*N* = 32)	*MVIS (N.kg* ^*−1*^ *)*	0.007 ± 0.13	−0.38 to 0.40
	*RFG (N.kg* ^*− 1*^ *.s* ^*− 1*^ *)*	0.23 ± 1.75	−3.01 to 3.48
Fallers (*N* = 16)	*MVIS (N.kg* ^*− 1*^ *)*	0.008 ± 0.16	− 0.28 to 0.29
	*RFG (N.kg* ^*− 1*^ *.s* ^*− 1*^ *)*	0.67 ± 1.48	−2.42 to 3.76
Non-fallers (*N* = 16)	*MVIS (N.kg* ^*− 1*^ *)*	0.006 ± 0.12	−0.48 to 0.44
	*RFG (N.kg* ^*− 1*^ *.s* ^*− 1*^ *)*	−0.21 ± 1.93	− 3.04 to 2.63

*SD* standard deviation, *LOA* limits of agreement, *MVIS* maximal voluntary isometric strength, *RFG* rate of force generation

### Comparison of hip MVIS and RFG between test-sessions in fallers and non-fallers

There were NS differences in hip abductor strength between sessions for both parameters (MVIS and RFG) for the whole group and both sub-groups. MVIS was significantly lower in the fallers compared to the non-fallers in both the first (fallers: 0.94 ± 0.32 N.kg^− 1^ vs. non-fallers: 1.22 ± 0.42 N.kg^− 1^, *p* = 0.0012) and second tests (fallers: 0.93 ± 0.28 N.kg^− 1^ vs. non-fallers: 1.21 ± 0.46 N.kg^− 1^, *p* = 0.0126). A similar result was found for the RFG in both the first (fallers: 6.75 ± 3.12 N.kg^− 1^.s^− 1^ vs. non-fallers 9.10 ± 2.75 N.kg^− 1^.s^− 1^, *p* = 0.0022) and second tests (fallers: 6.08 ± 3.24 N.kg^− 1^.s^− 1^ vs. non-fallers 9.31 ± 3.23 N.kg^− 1^.s^− 1^, *p* = 0.0001). In the scatterplots (Fig. 3, plots a and b), the values of the open dots (fallers) are lower (less strength) than the closed dots (non-fallers).

## Discussion

The aim of this work was to evaluate the reliability of a functional and user-friendly test to measure hip abductor strength in older people. This is particularly important since hip abductor strength is known to be associated with a risk of falls [4]. The results showed that testing hip strength in a standing position is feasible and rapid. All participants successfully completed the test in less than four minutes. Moreover, intra-rater reliability was good for the total sample as well as for both the faller and non-faller subgroups. As testing hip strength in a standing position is feasible, reliable, and better reflects real life situations than strength tests in supine or side-lying positions, we recommend this position for clinical practice.

The test-retest reliability for MVIS found in the present study was similar to that found by Wang et al. in supine position [29]. They evaluated the reliability of strength measurements using a HHD in 8 lower limb muscle groups in community-dwelling, older fallers. However, this study included only fallers without a comparison with non-fallers. In our study, ICC values for MVIS were better in non-fallers than fallers, while the intra-rater reliability for RFG was lower for the non-fallers than for fallers. Nevertheless, ICC values remained above 0.75 which is a good reliability and an appropriate threshold value for clinical use [28]. Our results and those of Wang et al. [29] showed that hip abductor strength can be measured reliably in older subjects at risk of falls both in supine and standing position.

Widler et al. assessed intra-rater reliability in young adults [12]. They compared intra-rater reliability in standing (ICC = 0.88), side-lying (ICC = 0.90), and supine positions (ICC = 0.82). They found the highest ICC in side-lying, although reliability was also good in the two other positions. The level of test-retest reliability found in the present study was higher than in the study by Widler et al. [12]. This difference might be due to more heterogenic strength values for our study sample, but also due to differences in the test position [21]. In the present study both forearms of the participants were supported on a high treatment table, while in the study by Widler et al. subjects stabilized themselves with only one hand [12]. The importance of stabilization on reliability has been confirmed by a recent study [16]. Awwad et al. [16] who assessed hip abductor strength in older adults in a standing position showed values close to our results. In contrast to our study their participants stabilized the pelvis against a wall and participants indicated to perceive fatigue in the opposite hip to that being tested [16]. This was probably due to the fact that standing leg was necessary to stabilize the pelvis in this position. Awwad et al. [16] further showed an increased intra-subject variability in strength values recorded in the standing position compared to the supine position (in which the pelvis was also stabilized against the wall). In our study the pelvis was not stabilized against a wall. Nevertheless, we found similar ICC’s to Awwad et al. and none of the patients complained of fatigue in the supporting leg. These facts support the use of the testing position described in the current study. In addition, our test has the advantage of being closer to different motor functions during which the pelvis is never stabilized.

Another explanation for the discrepancy between the study of Widler et al. [12] and our results could be the differences in the subjects’ ages. The greater strength of young subjects induced certainly greater difficulties to avoid compensations [15]. As the muscle strength in fallers was lower than in non-fallers, it is likely that fallers compensated less which might have increased reliability. In the supine position with belt-resisted dynamometer fixation during strength testing, Kramer et al. found higher test-retest reliability in older subjects (ICC > 0.98) than in young adults (ICC > 0.92) [15]. The authors attributed this ICC difference to the compensation during test but also to higher between-subject strength variation in older adults. In standing position, the intra-rater reliability of MVIS measured in older adults in our study was higher (ICC > 0.94) than the intra-rater reliability of MVIS in young healthy subjects of the study of Widler et al. (ICC > 0.88) [12]. The results of these studies corroborate the results of Kramer et al. who tested older and young adults in a side-lying position [15]. Among the study population fallers were eight years older than non-fallers. However, the ICC of hip abductor MVIS were similar in fallers and in non-fallers whereas higher ICC’s were found for the RFG measures in fallers (ICC = 0.93) than in non-fallers (ICC = 0.88). It might be that the RFG is more influenced by age than the MVIS and thus induces more heterogeneity and consequently a higher ICC values in RFG parameter. It is well known that movement slows down with advancing age which is thought to be partially strategic in that older adults give emphasis to movement accuracy at the cost of movement speed [30]. In addition, slower information processing due to an increase in neural noise and other synaptic changes may also affect the RFG [30]. Nevertheless, we cannot exclude that several other unknown factors playing a role in these differences.

In both the present study and the study by Widler et al. [12], the HHD was fixed. Several studies have shown that when a HHD is fixed externally, subjects generate higher magnitudes of peak force, and test-retest reliability is higher than for manual, examiner resisted HHD measurements [15, 31]. In our study, the HHD was fixed to the wall, thus the procedure was standardized, and no bias was created by variations in rater-force. Moreover, the procedure is faster when the HDD is fixed externally.

Comparison of the strength parameters showed that reliability was higher for MVIS than RFG. To our knowledge, no other study has evaluated the test-retest reliability of the RFG of the hip abductors in a standing position, despite the fact that RFG is an important parameter relating to dynamic balance control in older subjects [4, 17, 18]. Although the reliability of RFG was lower than MVIS, it was still good (ICC > 0.88). Thus, according to clinical recommendations by Portney and Watkins [28], this parameter can be used to evaluate hip abductor strength in clinical practice. We believe it is relevant to assess both MVIS and RFG since they are not correlated [20], and both are associated with an increased risk of falls in older women [4]. In clinical practice, the dynamometer usually directly shows MVIS, but not RFG. Thus, in the future, it would be useful to develop HHD tools with RFG values directly displayed on the screen facilitating the use by clinicians.

ICCs are highly dependent on the heterogeneity of the study sample while SEM, SDD and LOA are more appropriate to evaluate changes over time [32]. SEM values are also interesting to clinicians in terms of decision making, since they describe errors in the same units of measurement, and can be used to calculate SDD between two measurements [33]. In standing, the SEM for MVIS was very similar between fallers and non-fallers, however, the SDD was higher in fallers (respectively 32.7 and 20.3%). In whole group, the SDD of RFG was higher (44.3%) than the SDD of MVIS (25.2%). Mentiplay et al. showed the same results for young subjects measured in a supine position with a SDD of RFG of 34.65% and a SDD of MVIS of 20.23% [23]. The SDDs found in the present study were lower than the ones in our previous study that measured hip abductor strength in older adults in a side-lying position (RFG: 51%, MVIS: 32%) [13]. Thus, we recommend in clinical practice testing hip abductor strength in older persons at risk of falls in a standing position. Similarly to Widler et al. [12] and Awwad et al. [16], who evaluated maximal hip strength value in a standing position, Bland and Altman analysis for intra-rater reliability showed no systematic session effect for the MVIS. This suggests that *no learning effect* or fatigue occurred in the patients or raters and confirms the quality of our procedure without previous practical trials for this parameter. Nevertheless, Bland and Altman analysis showed somewhat higher mean between sessions differences for RFG, especially for the fallers, indicating a small bias, i.e. a small learning effect between sessions. The greater mean differences between session for RFG than for MVIS have also been mentioned by Mentiplay et al. [23]. The good intra-rater reliability and the small systematic effect which has previously been observed for MVIS in older people [16], the lower SDD observed in older persons at risk of falls in a standing position compared to the ones of older persons in a side-lying [13] and the feasibility highlight the interest of the clinical abductor test in standing position.

It should be noted that the present study only evaluated intra-rater reliability. In addition, the test position of participants was only verified visually without the use of precise sensors. However, this reflects clinical practice. In addition, the rater was not blinded to the faller or non-faller status of the participant. In this study, before tests, the subjects haven’t had a separate familiarization session. We decided for this procedure according to our pilot test as well as to avoid additional fatigue for these older subjects. Indeed, we think that fatigue is a more important bias than the lack of a familiarization session. However, we believe that the reliability could be improved for patients with low fatigue and who have had a practice trial, especially for RFG parameter. The MVIS and RFG parameters were assessed in the same trial, in order to avoid too many repetitions for the participants. This implicated a more complex test instruction (push hard and fast) which may have been difficult for some participants to achieve. Testing MVIS and RFG in two separate trials may produce more accurate results for the assessment of strength in an older population.

This study focused on immediate isometric hip abductor strength without considering endurance, which is an important parameter for activities of daily living. Moreover, it is well known that type 1 muscle fibers tend to degenerate in older people [34, 35]. Van Cant et al. [35] found good test-retest reliability for both isometric and isotonic hip abductor endurance in a side-lying position in young adults. Therefore, future studies should determine the reliability of testing both maximal isometric strength and endurance of the hip abductors in a standing position in older subjects.

## Conclusion

Assessment of hip abductor strength (MVIS and RFG) using a HHD in a standing position is feasible and reliable in older people at risk of falls. The test is easy to carry out in clinical practice.

The intra-rater reliability of abductor strength test with HDD seems associated to study population (age, pathology, …), testing position and testing procedure. Our results showed that hip abductor strength in older persons can reliably be measured in a standing position with the dynamometer fixed against a wall, pelvis frontal movement not restricted and an upper limb support on a treatment table to secure the patient. Moreover, assessment in a standing position is more functional than in supine or side-lying and provides a better evaluation of real-life activities in which falls occur. We thus recommend assessment of hip abductor strength in a standing position.

## Abbrevations

HHD: hand-held dynamometer
ICC_agreement_: Intraclass Correlation Coefficient Agreement
LOA: Limits of agreement
MMSE: Minimal Mental State Exam
MVIS: Maximum Voluntary Isometric Strength
NS: Non significant
RFG: Rate of Force Generation
SDD: Smallest detectable difference
SEM: Standard Error of Measurement
SPPB: Short Physical Performance Battery
TUG: Time Up and Go Test
